# A vicious cycle among cognitions and behaviors enhancing risk for eating disorders

**DOI:** 10.1186/s12888-017-1328-9

**Published:** 2017-04-28

**Authors:** Karolina Zarychta, Barbara Mullan, Magdalena Kruk, Aleksandra Luszczynska

**Affiliations:** 1 Psychology Department in Wroclaw, SWPS University of Social Sciences and Humanities in Wroclaw, 30b Ostrowskiego Street, PL-53-238 Wroclaw, Poland; 2 Health Psychology & Behavioural Medicine Research Group, School of Psychology and Speech Pathology, Faculty of Health Sciences, Curtin University, Bentley, WA Australia; 3 Trauma, Health, and Hazards Center, University of Colorado at Colorado Springs, 1861 Austin Bluffs Pkwy, Colorado Springs, CO 80918 USA

**Keywords:** Adolescence, Eating disorders risk factors, Transdiagnostic model, Non-clinical sample

## Abstract

**Background:**

Establishing the sequence in which risk factors for eating disorders (ED) emerge would enable more effective ED prevention. Thus, in our study we investigated reciprocal and indirect associations between three cognitive and behavioral ED determinants (appearance orientation, appearance worries, and dieting) emphasized in the transdiagnostic model of ED.

**Methods:**

Data were collected in a non-clinical group of adolescents at Time 1 (T1), and then 2-months (Time 2, T2)﻿﻿﻿ and 13-months later (Time 3, T3). Participants (*N* = 1260) aged 13–19 completed a questionnaire encompassing their nutrition behaviors, beliefs about appearance, health and well-being. Weight and height were measured objectively.

**Results:**

Higher levels of appearance orientation (T1) were associated with higher levels of appearance worries (T2) which in turn predicted dieting (T3). Dieting (T1) predicted higher levels of appearance orientation (T2) which in turn predicted higher levels of appearance worries (T3). Higher levels of appearance worries (T1) were associated with higher levels of appearance orientation (T2) which in turn predicted dieting (T3). Also, higher levels of appearance worries (T1) were associated with dieting (T2), and higher levels of appearance orientation (T3).

**Conclusions:**

The three transdiagnostic model variables formed a vicious cycle. Therefore, higher levels of one of ED determinants (appearance orientation, appearance worries or dieting) increase the likelihood of the elevated levels of two other ED determinants at follow-ups and thus enhances the risk for ED.

**Electronic supplementary material:**

The online version of this article (doi:10.1186/s12888-017-1328-9) contains supplementary material, which is available to authorized users.

## Background

Eating disorders (EDs) are the third most common chronic disorder in adolescence and their prevalence continues to increase, affecting more than 1 % of adolescent males and females worldwide [1, 2]. Symptoms of EDs that do not meet the diagnostic criteria [3, 4] may occur also in the general population of adolescents [5]. EDs are associated with severe or even life-threatening health effects [1]. Therefore, identification of risk factors for EDs and the order in which they operate is of key importance in the prevention of EDs and their consequences.

The onset of ED, its etiology and maintenance are hypothesized to depend on a set of cognitive factors highlighted in theoretical models and research. Cognitive models of EDs [6–8] focus primarily on the way people perceive their bodies, the contents of their thoughts, and perceptions of body weight and shape. The transdiagnostic model of EDs emphasizes that people who are preoccupied with body weight and shape, are also more vigilant and anxious about their body fat, and let their outer appearance affect their self-evaluations [8, 9]. This may lead to an excessive concentration on weight loss and to pathological behaviors (i.e. restrictive dieting). Dietary restriction may lead to underweight or compulsive overeating and compensatory behaviors, which in turn gives rise to even more preoccupation with body weight and shape [7]. Thus, the transdiagnostic model of EDs suggests a specific causal chain of effects, starting with preoccupation with body weight and shape, which leads to excessive self-control and body anxiety, which in turn predicts using various methods of weight loss, such as dieting.

Yet, research has not provided a longitudinal test of the transdiagnostic model, with the three cognitive-behavioral factors measured at separate time points and whether appearance orientation is indeed the trigger for the appearance worries which in turn is followed by restrictive dieting. Previous tests of the transdiagnostic model of EDs have applied a cross-sectional design [10–12] or have not tested alternative associations between these variables [13, 14]. Thus, we hypothesized that each of the three variables may be a starting point for the vicious cycle and that this variable may negatively impact either of the other two variables. Using a longitudinal design with three measurement points we aimed to examine the reciprocal associations between appearance orientation, appearance worries, and dieting in a non-clinical sample of adolescents. In particular, it was hypothesized that:

the indirect effect of appearance orientation (Time 1; T1) on dieting (Time 3; T3) would be mediated by appearance worries (Time 2, T2) (Hypothesis 1; H1);

the indirect effect of appearance orientation (T1) on appearance worries (T3) would be mediated by dieting (T2) (H2);

the indirect effect of appearance worries (T1) on dieting (T3) would be mediated by appearance orientation (T2) (H3);

the indirect effect of appearance worries (T1) on appearance orientation (T3) would be mediated by dieting (T2) (H4);

the indirect effect of dieting (T1) on appearance orientation (T3) would be mediated by appearance worries (T2) (H5).

the indirect effect of dieting (T1) on appearance worries (T3) would be mediated by appearance orientation (T2).

Analyzed variables were hypothesized to form a vicious cycle. As previous research established that sex [11, 14–16] and weight status or BMI [10, 11, 14, 17–19] may be relevant determinants of the cognitive and behavioral ED risk factors, all hypotheses were tested controlling for the effects of sex and body weight status (underweight, normal body weight, overweight, obesity) on the respective dependent variable. Additional analyses were conducted in order to test if participants’ sex may moderate the associations between the three key variables included in the models.

## Methods

### Participants

At T1, 1260 adolescents (41.7% girls) aged 13 to 19 (*M* = 16.38, *SD* = 0.80) took part in the study. Their body mass index was ranging from 15.49 to 41.26 (*M* = 21.97, *SD* = 3.18) with 11 (0.9%) respondents being underweight, 1000 (79.4%) with normal body weight, 204 (16.2%) with overweight, and 45 (3.6%) with obesity. Two months later (at T2) 1059 adolescents provided their data. At T3 (13 months later), a total of 935 (51.7% girls) adolescents aged 14 to 20 years old (*M* = 17.40, *SD* = 0.90) with BMIs ranging from 15.66 to 39.26 (*M* = 21.96, *SD* = 3.19) participated in the study (for more details about the sample see [20]). All participants were white (general population of Poland is 96% white).

The total attrition rate was 37.4%. Regression method (maximum likelihood procedure) was used to impute missing data from participants who dropped out at T2 or T3. Thus, data collected from *N* = 1260 adolescents (41.7% girls) aged 13 to 19 years old (*M* = 16.38, *SD* = 0.80) with BMIs ranging from 15.49 to 41.26 (*M* = 21.97, *SD* = 3.18) were included in the analyses. Demographic and clinical characteristics of two sex groups are presented in Additional file 1.

### Procedure

Data were collected three times with 2- and 13-months follow ups. The intervals between the assessment points were chosen to represent the short-term and long-term associations between the study variables. Thus the intervals often used in clinical psychology research for short-term (up to 3 months; e.g. [21, 22]) and long-term follow-ups (at least 12 months; e.g. [23]) were applied.

Adolescents from sixteen public middle and high schools in Central and Eastern Poland took part in the study. All respondents lived with their parents (98.9%) or other legal guardians (1.1%) at T1 and T2. Participants and parents of those younger than 18 years old provided written informed consent prior to the data collection. Informed consent obtained from the parent and the participants, and being older than 12 years old and younger than 19 at T1 were the only inclusions criteria. There were no exclusion criteria. Individuals were informed about the aims and the procedure of the study. Once the consent was provided, adolescents got their personal codes, filled in a questionnaire regarding nutrition behaviors, beliefs about appearance, health and well-being, and have their biometric measures taken. This procedure was repeated at the following measurement points. Several attempts were made to reduce drop-out rate, but attrition was still relatively high, mainly because of completing secondary education, changing or dropping out of school by adolescents (see the Attrition analysis in the Results section). The study was approved by the Institutional Review Board at the first author’s university. The procedures of the study were described in more detail in a paper by Zarychta et al..

### Materials

Means, standard deviations, and reliability coefficients are presented in Table 1.

**Table 1:** Descriptive statistics, reliability, and correlations between the study variables at T1, T2 and T3 (*N* = 1260)

	*M (SD)*	*α*	T2 appearance orientation	T3 appearance orientation	T1 appearance worries	T2 appearance worries	T3 appearance worries	T1 diet	T2 diet	T3 diet	T1 weight status	T2 weight status	T3 weight status	T1 age	Sex
T1 Appearance orientation	3.74 (0.68)	0.63	0.69***	0.48***	0.40***	0.36***	0.21***	0.19***	0.20***	0.09**	0.06*	0.05	0.03	0.08**	0.32***
T2 Appearance orientation	2.39 (0.64)	0.61		0.44***	0.31***	0.36***	0.17***	0.18***	0.11***	0.002	0.06*	0.06*	0.03	0.10***	0.34***
T3 Appearance orientation	2.56 (0.57)	0.50			0.21***	0.21***	0.26***	0.12***	0.04	- 0.02	0.04	0.04	0.04	0.11***	0.21***
T1 Appearance worries	2.80 (1.24)	0.51				0.66***	0.41***	0.46***	0.30***	0.23***	- 0.18***	- 0.19***	- 0.15***	0.09**	0.33***
T2 Appearance worries	3.11 (1.09)	0.45					0.42***	0.38***	0.42***	0.18***	- 0.13***	- 0.12***	- 0.10***	0.03	0.28***
T3 Appearance worries	3.13 (1.05)	0.34						0.21***	0.17***	0.39***	- 0.11***	- 0.09**	- 0.11***	0.04	0.17***
T1 Diet	1.74 (1.25)								0.44***	0.24***	- 0.18***	- 0.20***	- 0.16***	0.05^†^	0.20***
T2 Diet	3.93 (1.31)									0.27***	- 0.21***	- 0.21***	- 0.17***	- 0.02	0.13***
T3 Diet	3.76 (1.28)										- 0.13***	- 0.12***	- 0.15***	0.02	0.03
T1 weight status	1.22 (0.51)											0.83***	0.68***	- 0.03	0.04
T2 weight status	1.24 (0.53)												0.71***	- 0.001	0.05^†^
T3 weight status	1.19 (0.49)													0.03	0.06*
T1 Age	16.38 (0.80)														- 0.001
Sex															

***p < 0.001; **p < 0.01; *p < 0.05; ^†^ *p* < 0.1; weight status: 0 – underweight, 1 – normal, 2 – overweight, 3 – obesity; *T1* Time 1, baseline, *T2* Time 2, 2-month follow-up, *T3* Time 3, 13-month follow-up

#### Appearance orientation (T1, T2, and T3)

The appearance orientation scale consisted of 12 items, based on The Multidimensional Body-Self Relations Questionnaire’s Appearance Orientation Subscale (MBSRQ; 24). The construct considers the extent of investment in one’s appearance with high scores indicating higher importance and more attention paid to appearance, and more frequent engaging in grooming behaviors. Respondents were asked to read twelve statements (e.g., “Before going out in public, I always notice how I look”, “I check my appearance in a mirror whenever I can” and “I take special care with my hair grooming”) and decide how much each statement pertains to them. The responses ranged from 1 (definitely disagree) to 5 (definitely agree).

#### Appearance worries (T1, T2, and T3)

Two items were used to assess appearance worries, based on MBSRQ’s Overweight Preoccupation Scale [24]. The construct reflects fat anxiety and weight vigilance with high scores indicating more vigilance and anxiety about body fat. Respondents read two statements “I constantly worry about being or becoming fat” and “I am very conscious of even small changes in my weight”, and decide how much each statement pertains to them. The responses ranged from 1 (definitely disagree) to 5 (definitely agree).

#### Dieting (T1, T2, and T3)

In line with previous research [25], the measurement of dietary restrictions was based on one item. Participants responded to an item based on MBSRQ [24]: “I am on a weight-loss diet”. The responses ranged from 1 (definitely disagree) to 5 (definitely agree).

#### Weight status (T1 and T3)

To assess body weight and height standard medically-approved telescopic height measuring rods and floor scales (scale type: BF-100 or BF-25) were used. With WHO AnthroPlus macro [26] age- and sex specific BMI percentiles were calculated (for a more details see [27]). BMIs were coded onto three weight status categories based on *SD* cut-offs (0 – underweight [less than or equal to 2 *SD*], 1 – normal weight, 2 – overweight or obesity [greater than or equal to 1 *SD*]) based on WHO growth reference [28].

### Data analysis

**Fig. 1:**
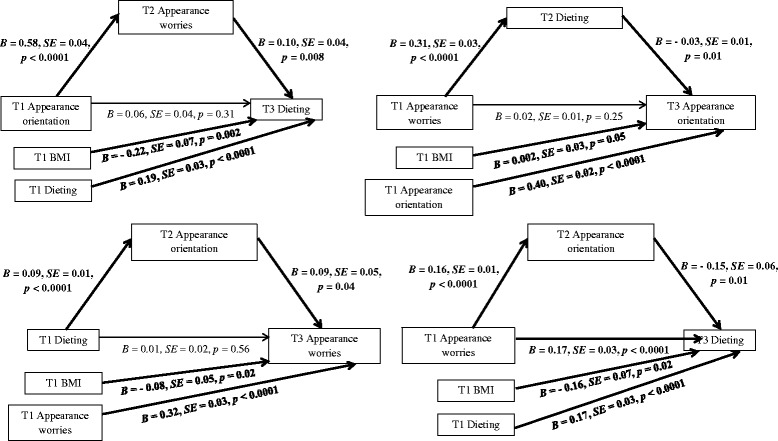
The mediating effects of appearance orientation, appearance worries, and dieting. Note. T1 = Time 1, baseline; T2 = Time 2, 2-month follow-up, T3 = Time 3, 13-month follow-up; H = Hypothesis. Findings referring to H1 are presented in upper left panel, findings referring to H3 and H4 are presented in upper and bottom right panels, findings referring to H6 are presented in bottom left panel. Paths marked in bold represent significant associations

**Fig. 2:**
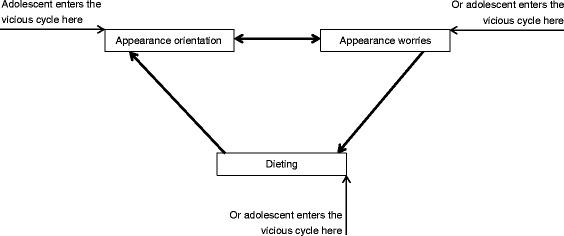
Vicious cycle formed by tested variables

All analyses were made with SPSS version 23. Mediation analyses were performed to test whether there was a reciprocal relation between appearance orientation, appearance worries, and dieting, with the use of PROCESS macro with 5000 bootstraps [29]. The macro permits for conducting multiple mediator regression analysis, accounting for the covariates (T1 weight status and the respective dependent variable measured at T1). Moreover, PROCESS allows for testing the significance of indirect effects. Two types of coefficients present the results of the analyses: (1) a regression coefficient for each parameter (see Fig. 1) and (2) the indirect effect coefficient (*B*) for each indirect pathway between the independent variable (T1 appearance orientation, T1 appearance worries or T1 dieting) and the dependent variable (T3 appearance orientation, T3 appearance worries or T3 dieting), accounting for respective mediators and covariates (see Fig. 1 and Table 2). Participants’ body weight status was controlled in each analysis. This approach to testing bidirectional relationship and causal order of variables was recommended in research on methodology and statistical advancement [30], and applied in eating disorder studies [5, 31–33].

**Table 2:** Reciprocal effects of appearance orientation, appearance worries, and dieting

Indirect effects pathways		*B*	*SE*	BC 95% CI	
				Lower	Higher
*Testing the indirect effect of appearance orientation*					
*Model 1*	**Appearance orientation T1 ➔ Appearance worries T2 ➔ Dieting T3 (H1)**	**0.06**	**0.02**	**0.02**	**0.10**
	Appearance orientation T1 ➔ Dieting T2 ➔ Appearance worries T3 (H2)	0.01	0.01	- 0.01	0.03
*Testing the indirect effect of appearance worries*					
*Model 2*	**Appearance worries T1 ➔ Appearance orientation T2 ➔ Dieting T3 (H3)**	**- 0.02**	**0.009**	**- 0.04**	**- 0.01**
	**Appearance worries T1 ➔ Dieting T2 ➔ Appearance orientation T3 (H4)**	**- 0.01**	**0.004**	**- 0.02**	**- 0.001**
*Testing the indirect effect of dieting*					
*Model 3*	Dieting T1 ➔ Appearance worries T2 ➔ Appearance orientation T3 (H5)	0.01	0.01	- 0.004	0.02
	**Dieting T1 ➔ Appearance orientation T2 ➔ Appearance worries T3 (H6)**	**0.01**	**0.004**	**0.001**	**0.02**

Values of indirect effect coefficient (*B*) presented in bold are significant. Each bootstrap was based on 5000 repetitions. Bias corrected (BC) confidence intervals (CI) that do not include zero indicate a significant indirect effect. *T1* Time 1, baseline, *T2* Time 2, 2-month follow-up, *T3* Time 3, 13-month follow-up, *H* Hypothesis. Significant coefficients are marked in bold

Additionally, moderated mediation analyses were conducted for each hypothesized model using PROCESS macro (Model 59). In particular, these analyses tested if the associations between (1) the independent variable and the mediator, (2) the mediator and the dependent variable, and (3) the independent and the dependent variables are moderated by participants’ sex.

In the current study the independent variables (IV) in respective analyses were either appearance orientation (T1) (Model 1; H1 and H2), or appearance worries (T1) (Model 2; H3 and H4), or dieting (T1) (Model 3, H5 and H6); the mediators were these variables measured at T2, and the dependent variables (DV) were measured at T3. As suggested by MacKinnon [34], the independent variables, the mediators, and the dependent variables in the respective equations were measured at different time points (T1, T2, and T3) in order to establish temporal precedence. Missing data for the study variables measured at T1 (due to lack of the answers in completed questionnaires), T2 and T3 (due to the dropping-out) were imputed with multiple imputation (regression method), which is an effective way of treating data, even if up to 50% is missing [35]. A dataset created by averaging five data sets with imputed values was used in the analyses. Overall, a total of 38.2% of data were missing across T1, T2, and T3, with only 6.7% of missings in data obtained by completers. The attrition analysis is presented below.

## Results

### Attrition analysis

Completers did not differ from those who dropped out at T2 or T3 in terms of appearance orientation, appearance worries, dieting, and weight status, all *F*s < 2.29, *p*s > 0.13, or sex, χ2 (1) = 2.73, *p* = 0.13. Dropouts and completers differed in terms of age, *F* (1, 1259) = 3.99, *p* = 0.05 with dropouts being slightly older (*M* = 16.46, *SD* = 0.71) than completers (*M* = 16.36, *SD* = 0.83, Cohen’s *d* = 0.13 [95% CI: 0.08 to 0.17]).

### Results of correlation analysis

Correlations between study variables for the total sample (*N* = 1260) are presented in Table 1. Higher levels of appearance orientation (T1, T2, and T3) were correlated with higher levels of appearance worries (T1, T2, and T3), and dieting (T1, T2, and T3). All three variables were more likely to be observed among female participants. Higher levels of appearance worries (T1, T2, and T3) were associated with dieting (T1, T2, and T3) and with lower weight status (T1, T2, and T3). Moreover, adolescents’ dieting behaviors (T1, T2, and T3) were correlated with adolescents’ lower weight status (T1, T2, and T3).

### Testing the indirect effect of appearance orientation, with either appearance worries (H1) or dieting (H2) operating as the mediators

The results of the mediation analyses for Model 1 (see Table 2) showed that the effect of appearance orientation (T1) on dieting (T3) was mediated by appearance worries (T2), but there was no significant indirect effect of appearance orientation (T1) on appearance worries (T3) through dieting (T2). Adolescents who were more appearance orientated (T1) were also more worried about their appearance at T2 which in turn predicted dieting more often at T3. Significant direct associations between IV (T1), mediator (T2), and DV (T3) are presented in Fig. 1 (upper right). Both analyses were conducted with controlling for weight status (i.e., underweight, normal body weight, overweight or obesity) and the dependent variable measured at T1. Additional moderation analyses, assuming that the associations between appearance worries, appearance orientation, and dieting were moderated by sex, indicated that these associations were the same for girls and boys.

### Testing the indirect effect of appearance worries, with either appearance orientation (H3) or dieting (H4) operating as the mediators

The results showed that both mediation analyses and respective indirect effects for Model 2 (see Table 2) were significant. Thus, the effect of appearance worries (T1) on dieting (T3) was mediated by appearance orientation (T2), and the effect of appearance worries (T1) on appearance orientation (T3) was mediated by dieting (T2). Adolescents more worried about their appearance (T1) were also more appearance oriented at T2, which in turn predicted more frequent dieting at T3. Moreover, adolescents more worried about their appearance (T1) were also dieting more often at T2, which in turn predicted higher levels of appearance orientation at T3. Significant direct associations between IV (T1), mediators (T2), and DVs (T3) are presented in Fig. 1 (upper and bottom left). Both analyses were conducted with controlling for weight status (i.e., underweight, normal body weight, overweight, obesity) and the dependent variable measured at T1. Additional moderation analyses were conducted for Model 2. The analyses assumed that the associations between appearance worries, appearance orientation, and dieting were moderated by sex, indicated that these associations were the same for girls and boys.

### Testing the indirect effect of dieting with either appearance worries (H5) or appearance orientation (H6) acting as the mediators

The results of mediation analyses for Model 3 (see Table 2) showed that the effect of dieting (T1) on appearance orientation (T3) was mediated by appearance worries (T2), but no significant indirect effect of dieting (T1) on appearance worries (T3) through appearance orientation (T2) was found. Thus, adolescents who were dieting at T1 were more worried about their appearance at T2 which in turn predicted higher appearance orientation at T3. Significant direct associations between IV (T1), mediator (T2), and DV (T3) are presented in Fig. 1 (bottom right). Both analyses were conducted with controlling for weight status and the dependent variable measured at T1. Additional moderation analyses conducted for Model 3 were conducted. It was tested if the associations between appearance worries, appearance orientation, and dieting were moderated by sex. No significant effects were found indicating that the associations were the same for girls and boys.

Ultimately, the results obtained in the analyses conducted for Hypotheses 1–6 indicate that the three variables form a specific vicious cycle. The cycle is presented in Fig. 2.

## Discussion

By using a longitudinal design this study provides novel evidence for the order of the associations between appearance orientation, appearance worries, and dieting in the non-clinical group of adolescents. The results show that appearance orientation, appearance worries, and dieting are highly intertwined. In particular, higher appearance orientation at T1 predicted more appearance worries at T2 which in turn predicted dieting at T3. Moreover, appearance worries at T1 were associated with appearance orientation at T2 which predicted dieting at T3. Higher levels of appearance worries at T1 were also associated with dieting at T2 which predicted being more appearance orientated thinking at T3. Dieting at T1 was associated with higher levels of appearance worries at T2 which in turn predicted higher levels of appearance orientation at T3. Thus, appearance orientation, appearance worries, and dieting [7], were all individually the starting points for specific sequences of tested variables.

In contrast to previous cross-sectional research [10]; or research which explored direct associations between variables presented in the transdiagnostic model of ED [13, 14], this research permitted a more thorough testing of the order in which appearance orientation, appearance worries, and dieting may operate. Thus our study offers a novel evidence which verifies a part of the transdiagnostic model of ED [7]. Importantly our research expands on previous research and shows that these associations are true across the range of body weight status and in a large sample of both male and female participants.

Overall, we tested the order hypothesized in the transdiagnostic model of ED [7] and compared it with five alternative orders, three of which were confirmed. Our study provides a strong test for the order of associations between the three variables derived from the transdiagnostic model. Thus, our findings provide an argument for revisiting the model [7] and assuming that some of the uni-directional associations may be bi-directional. First, the model assumes the three variables operate in a specific order (e.g. from appearance orientation through appearance worries to restrictive dieting). We confirmed that these three variables indeed operate in this order. However, the findings point out that the order of the associations may be better represented if a vicious cycle between the three studied variables is assumed. In particular, the sequence “appearance orientation ➔ appearance worries ➔ dieting” is true whichever of the three variables is the starting point (see Fig. 1). In other words the model may need an additional path leading from dieting to appearance orientation (see Fig. 2). Second, we found that the variables such as appearance worries and dieting, which according to the transdiagnostic model [7] are assumed to be the mediators or the consequences of the cognitive processes constituting risk factors for ED, may also form a starting point (cf. Fig. 1). Thus, the model may need to be revised in order to highlight the fact that no matter the starting point in the chain, that is appearance orientation, appearance worries, or dieting, the other two factors will follow in respective order (see Fig. 2). Third, we found evidence suggesting that the hypothesized chain “appearance orientation ➔ appearance worries ➔ dieting” may also have a different order, i.e. appearance worries may exacerbate appearance orientation (see Fig. 1). Thus, suggesting a bi-directional association between the appearance orientation and appearance worries (see Fig. 2).

There were some limitations to the study, the main one being the high attrition rate. The results may be also affected by the specificity of the group, i.e. a non-clinical group of adolescents. The aim of this study, however, was to provide insight into potential mechanisms of developing ED. Thus, a clinical population with manifest ED would not have been appropriate to answer this research question. But yet, participants were not screened for ED symptoms or that they have met the diagnostic criteria for ED over the course of the study. Therefore, the conclusions may refer to a general population, which probably includes adolescents with and without ED symptoms. Also, it is impossible to say how many of participants will manifest clinically significant ED symptoms in the future and what goes with it, if the causal associations and the vicious cycle found in the study can lead to such symptoms. We did include adolescents’ weight status as covariant since it was found to be one of the relevant ED risk factors, but future research might also control for adolescents’ BMIs. Further, beta coefficients obtained in our study suggest weak associations between the variables and thus the result should be interpreted with caution. Moreover, it is possible that social desirability affected the participants’ responses. While the single-item measurement of dieting was in line with the approach used in previous research [25], it may have limited reliability, and future research could consider alternatives. Finally, we tested only a part of the transdiagnostic model and did not account for other factors included in the model, such as clinical perfectionism, mood intolerance, interpersonal problems, core low self-esteem, binge eating and compensatory behaviors, under-eating and low weight [7]. Future research should apply longitudinal design with more data collection points and include the remaining variables from the transdiagnostic model, explaining the formation and maintenance of ED symptoms.

## Conclusions

In conclusion, the results of this study specify the cognitive factors emphasized in the transdiagnostic model of ED [7]. Our findings suggest that appearance orientation, appearance worries, and dieting are reciprocally associated. These results indicate that the inclusion of cognitive factors such as appearance orientation and appearance worries, and behavioral symptoms of ED such as restrictive dieting in most treatment and prevention programs of ED is justified and should continue. Our findings, however, suggest that ED prevention or treatment programs might target not only to adolescents from clinical samples or with certain body weight status (e.g. underweight or overweight) but also adolescents who are at risk for dieting restrictively, who are highly appearance orientated or highly worried about their appearance. Such prevention programs may be useful for general population because the cognitive-behavioral patterns found in the present study in a sample drawn from the general population are similar to these present in clinical samples.

## Additional file

Additional file 1: Demographic and clinical characteristics of two gender groups. Note. ****p* < 0.001; ***p* < 0.01; **p* < 0.05; ^†^ *p* < 0.1; T1 = Time 1, baseline; T2 = Time 2, 2-month follow-up; T3 = Time 3, 13-month follow-up; BMI = body mass index. (DOCX 34 kb)

## Abbreviations

APA: American Psychiatric Association
BMI: Body Mass Index
ED: Eating disorders
H1: Hypothesis 1
H2: Hypothesis 2
H3: Hypothesis 3
MBSRQ: The Multidimensional Body-Self Relations Questionnaire’s Appearance Orientation Subscale
T1: Time 1
T2: Time 2
T3: Time 3
WHO: World Health Organization
