# Supplementary material for: A vicious cycle among cognitions and behaviors enhancing risk for eating disorders
Source: BMC Psychiatry. 2017 Apr 28;17:154. doi: 10.1186/s12888-017-1328-9 (PMC5410016; doi:10.1186/s12888-017-1328-9)
Supplement: Additional file 1: — Demographic and clinical characteristics of two gender groups. Note. ***p < 0.001; **p < 0.01; *p < 0.05; † p < 0.1; T1 = Time 1, baseline; T2 = Time 2, 2-month follow-up; T3 = Time 3, 13-month follow-up; BMI = body mass index. (DOCX 34 kb) [file 12888_2017_1328_MOESM1_ESM.docx]

Additional file 1. Demographic and clinical characteristics of two gender groups. **Note.** *** *p* < 0.001; ** *p* < 0.01; * *p* < 0.05; † *p* < 0.1; T1 = Time 1, baseline; T2 = Time 2, 2-month follow-up; T3 = Time 3, 13-month follow-up; BMI = body mass index.

|  | *Male* | | *Female* | |  |
| --- | --- | --- | --- | --- | --- |
|  | *M* | *SD* | *M* | *SD* | *t-test* |
| T1 Appearance orientation | 2.08 | 0.62 | 2.52 | 0.67 | - 11.85 |
| T2 Appearance orientation | 2.21 | 0.60 | 2.65 | 0.59 | - 12.70 |
| T3 Appearance orientation | 2.46 | 0.54 | 2.70 | 0.58 | - 7.46 |
| T1 Appearance worries | 2.86 | 1.21 | 3.69 | 1.13 | - 12.35* |
| T2 Appearance worries | 2.85 | 1.06 | 3.48 | 1.01 | - 10.49† |
| T3 Appearance worries | 2.97 | 1.05 | 3.34 | 1.00 | - 6.18 |
| T1 Diet | 4.05 | 1.36 | 4.55 | 1.03 | - 7.36** |
| T2 Diet | 3.78 | 1.38 | 4.14 | 1.17 | - 4.92* |
| T3 Diet | 3.73 | 1.28 | 3.81 | 1.27 | - 1.07 |
| T1 BMI | 21.70 | 3.30 | 22.36 | 2.96 | - 3.67*** |
| T2 BMI | 21.65 | 3.26 | 22.37 | 3.20 | - 3.86** |
| T3 BMI | 21.70 | 3.30 | 22.31 | 2.72 | - 3.67** |
| T1 Age | 16.39 | 0.69 | 16.38 | 0.94 | - 0.03 |
| T2 Age | 16.62 | 0.79 | 16.65 | 1.05 | - 0.54 |
| T3 Age | 17.36 | 0.80 | 17.46 | 1.03 | - 1.86 |
